# The monoacylglycerol acyltransferase pathway contributes to triacylglycerol synthesis in HepG2 cells

**DOI:** 10.1038/s41598-022-08946-y

**Published:** 2022-03-23

**Authors:** Pamela J. McFie, Apurv Patel, Scot J. Stone

**Affiliations:** Department of Biochemistry, Microbiology and Immunology, University of Saskatchewan, 107 Wiggins Road, Saskatoon, SK S7N 5E5 Canada

**Keywords:** Glycerides, Fat metabolism, Lipids

## Abstract

The monoacylglycerol acyltransferase (MGAT) pathway has a well-established role in the small intestine where it facilitates the absorption of dietary fat. In enterocytes, MGAT participates in the resynthesis of triacylglycerol using substrates (monoacylglycerol and fatty acids) generated in the gut lumen from the breakdown of triacylglycerol consumed in the diet. MGAT activity is also present in the liver, but its role in triacylglycerol metabolism in this tissue remains unclear. The predominant MGAT isoforms present in human liver appear to be MGAT2 and MGAT3. The objective of this study was to use selective small molecule inhibitors of MGAT2 and MGAT3 to determine the contributions of these enzymes to triacylglycerol production in liver cells. We found that pharmacological inhibition of either enzyme had no effect on TG mass in HepG2 cells but did alter lipid droplet size and number. Inhibition of MGAT2 did result in decreased DG and TG synthesis and TG secretion. Interestingly, MGAT2 preferentially utilized 2-monoacylglycerol derived from free glycerol and not from exogenously added 2-monoacylglycerol. In contrast, inhibition of MGAT3 had very little effect on TG metabolism in HepG2 cells. Additionally, we demonstrated that the MGAT activity of DGAT1 only makes a minor contribution to TG synthesis in intact HepG2 cells. Our data demonstrated that the MGAT pathway has a role in hepatic lipid metabolism with MGAT2, more so than MGAT3, contributing to TG synthesis and secretion.

## Introduction

The liver plays a central role in maintaining lipid homeostasis. Although the liver processes large amounts of fatty acids daily, triacylglycerol (TG) levels in this tissue are relatively low^1^. This low level of hepatic TG is maintained by a balance between fatty acid uptake from the circulation and their de novo lipogenesis and fatty acid oxidation and their secretion into the circulation as TG in very low-density lipoproteins (VLDL). Transient increases in liver TG can occur during fasting due to increased fatty acid availability caused by the mobilization of TG stored in adipose tissue^2^. A chronic accumulation of TG in the liver, caused by an imbalance between fatty acid acquisition and their disposal, can result in non-alcoholic fatty liver disease^3^. Non-alcoholic fatty liver disease is the most prevalent liver disease globally effecting 25% of the adult population^4^.

In most tissues, including the liver, TG biosynthesis can occur via the glycerol 3-phosphate (Kennedy) pathway where three fatty acids are sequentially esterified to a glycerol backbone via a series of four biochemical reactions^5–7^. Here, glycerol 3-phosphate, derived either from glycolysis or by the phosphorylation of glycerol by glycerol kinase, undergoes two sequential acylation reactions first generating 1-acylglycerol 3-phosphate and then phosphatidate. Phosphatidate is subsequently dephosphorylated by the phosphatidate phosphatase/transcriptional co-activator, lipin, producing 1,2-diacylglycerol (DG)^8,9^. In the fourth and final reaction of the pathway, DG is acylated by acyl-CoA:1,2-diacylglycerol acyltransferase (DGAT) producing TG that can be stored in cytosolic lipid droplets or secreted as a component of VLDL. Two forms of DGAT have been identified – DGAT1 and DGAT2^10,11^. These enzymes have no sequence homology and belong to distinct gene families^12^. In the liver, DGAT1 synthesizes a pool of TG whose fatty acids undergo oxidation^13^. In contrast, DGAT2 synthesizes TG that are secreted as VLDL.

In the small intestine, ~ 75% of TG synthesis occurs via the monoacylglycerol acyltransferase (MGAT) pathway^14,15^. The MGAT pathway plays a prominent role in dietary fat absorption as dietary TG are too hydrophobic to be absorbed from the small intestine intact. Instead, lipases released into the lumen of the small intestine hydrolyze TG to free fatty acids and 2-monoacylglycerol (2-MG) which are then taken up by intestinal epithelial cells where they are used as substrates to resynthesize TG. MGAT catalyzes the acyl-CoA-dependent re-esterification of 2-MG to 1,2-DG in enterocytes, which is used by DGAT enzymes to produce TG^14,16,17^. These TG are then stored in cytosolic lipid droplets or secreted into the circulation as a component of TG-rich chylomicrons via the lymph^18^.

Three MGAT isoforms (MGAT1, MGAT2 and MGAT3) have been identified based on their extensive homology to DGAT2^19–22^. Each isoform is encoded by a distinct *MOGAT* gene (*MOGAT1*, *MOGAT2* and *MOGAT3*) which are expressed in a species-specific manner^23,24^. In humans, *MOGAT2* and *MOGAT3* are both expressed in the small intestine. However, only MGAT2 has been shown to participate in intestinal lipid metabolism^25,26^. A role for MGAT3 in dietary fat absorption has yet to be demonstrated.

Other tissues, such as the liver, have historically been reported to possess a low level of MGAT activity^27–30^. Consequently, there is limited information available regarding the contribution of the MGAT pathway to hepatic lipid metabolism. More recent experiments have demonstrated that human liver in fact has robust MGAT activity, although it is not as high as in the small intestine^19,20,23^. Interestingly, all three *MOGAT* genes are expressed in human liver, while only *Mogat1* and *Mogat2* are expressed in mouse liver^21,23^. The role of MGAT1 in hepatic TG metabolism is not clear. Global or liver-specific disruption of *Mogat1* in mice had no effect on hepatic MGAT activity or TG levels^31^. Global disruption of *Mogat2* in mice results in an improved metabolic phenotype, including resistance to diet-induced obesity and protection against hepatic steatosis^25^. However, how much of this phenotype is due to the absence of MGAT2 in the liver is not known. Intestine-specific deletion of *Mogat2* in mice was also protective against hepatic steatosis while expression of *Mogat2* only in the small intestine of global *Mogat2*-deficicent mice caused accumulation of TG in the liver^26,32^. Because MGAT2 appears to be in low abundance in the liver, it was proposed that it was a minor contributor to MGAT activity in this tissue^23^. However, mice treated chronically with an MGAT2 inhibitor had reduced DG and TG synthesis in the liver^33^.

MGAT3 is the least studied MGAT isoform. MGAT3 shares more sequence similarity with DGAT2, than to either MGAT1 or MGAT2^21,22^. MGAT3 also possesses both MGAT and DGAT activities suggesting that it could function as a TG synthase^19,20,22–24^. Its in vivo function has not been determined as *Mogat3* in mice is a pseudogene and would not encode a functional protein^24^. Other appropriate animal models are also lacking. In this study, we tested the hypothesis that MGAT2 and/or MGAT3 can contribute to TG synthesis and secretion in the liver via the MGAT pathway. To test this, we inhibited MGAT2 and MGAT3 in HepG2 cells with highly selective small-molecule inhibitors and examined the effects on lipid metabolism^34,35^. We found that MGAT2, much more so than MGAT3, contributed to hepatic TG synthesis and secretion.

## Results

### MGAT2 and MGAT3 are minor contributors to total in vitro MGAT activity in HepG2 cells

**Figure 1:**
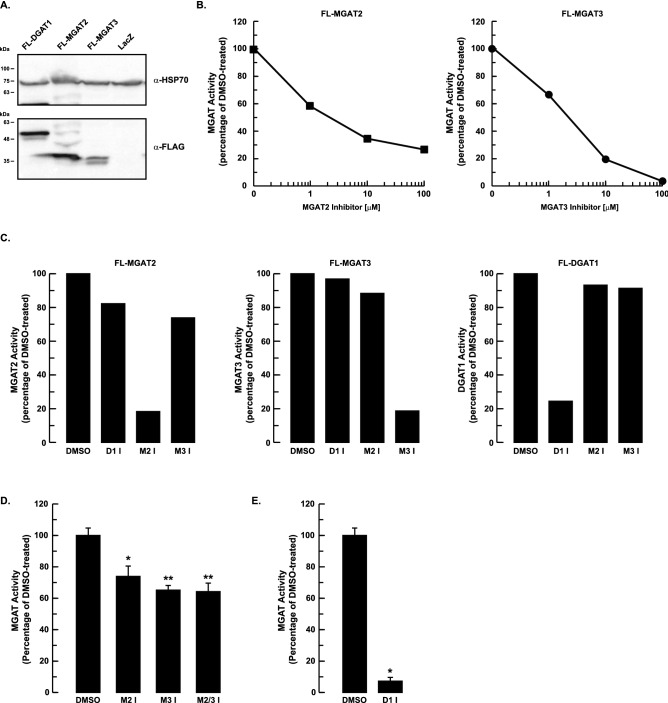
Pharmacological inhibition of MGAT2, MGAT3 and DGAT1. (**A**) Overexpression of FL-MGAT2, FL-MGAT3 and FL-DGAT1 in HEK-293T cells. Crude mitochondrial membrane fractions isolated from HEK-293T cells expressing FL-MGAT2, FL-MGAT3 or FL-DGAT1 were separated by SDS-PAGE and immunoblotted with anti-FLAG (α-FLAG) and anti-mitochondrial HSP70 (α-HSP70) antibodies. An uncropped image is shown in Supplementary Fig. 1. (**B**) The samples described in (**A**) were preincubated for 30 min with either the MGAT2 or MGAT3 inhibitors at the indicated concentrations. MGAT activity was determined and compared to control (DMSO-treated) samples. (**C**) Crude mitochondrial membrane fractions from HEK-293T cells expressing FL-MGAT2, FL-MGAT3 or FL-DGAT1 were preincubated for 30 min with either 25 µM MGAT2, 50 µM MGAT3 or 5 µM DGAT1 inhibitors. MGAT activity was determined and compared to control (DMSO-treated) samples. (**D**) Crude mitochondrial membrane fractions from HepG2 cells were pre-incubated with the MGAT inhibitors (10 µM) as described in Fig. 1C, separately or together. MGAT activity was determined and was normalized to control (DMSO-treated) cells. *p < 0.01 and **p < 0.001 *versus* DMSO-treated cells. (**E**) Inhibition of DGAT1 reduces MGAT activity in HepG2 cells. Crude mitochondrial membranes from HepG2 cells were pre-incubated with the DGAT1 inhibitor (2 µM final concentration) for 1 h. MGAT activity was determined and was normalized to control (DMSO-treated) samples. *p < 0.0001 versus DMSO-treated cells (unpaired two-tailed Student’s *t* test). D1 I, DGAT1 inhibitor; M2 I, MGAT2 inhibitor; M3 I, MGAT3 inhibitor; M2/3 I, MGAT2 and MGAT3 inhibitors.

The relative contributions of MGAT2 and MGAT3 to hepatic lipid metabolism are unknown. To probe the role that each enzyme has in TG synthesis and secretion in HepG2 cells we utilized the highly selective MGAT2 and MGAT3 small molecule inhibitors, Cpd24d and PF-06471553, respectively, to attenuate MGAT activity^34,35^. We first confirmed that these compounds effectively inhibited MGAT activity. When crude mitochondrial membrane fractions isolated from HEK-293T cells overexpressing either FLAG-tagged MGAT2 (FL-MGAT2) or FLAG-tagged MGAT3 (FL-MGAT3) were incubated with the MGAT2 or MGAT3 inhibitors, respectively, in vitro MGAT activities of both enzymes were reduced in a dose-dependant manner (Fig. 1A,B). Crude mitochondrial membranes were used for these enzyme assays instead of microsomes because MGAT2 and MGAT3 are enriched in this membrane fraction, which contains both mitochondria and mitochondrial-associated ER membranes^36–38^.

These MGAT inhibitors have been reported to be highly selective for their respective targets which we have confirmed^34,35^. The MGAT2 inhibitor reduced MGAT2 activity by ~ 80% and had a negligible effect on MGAT3 and DGAT1 activities (Fig. 1C). Similar results were obtained for the MGAT3 inhibitor and for PF-04620110, a DGAT1 inhibitor^39^.

We then determined if the MGAT2 and/or MGAT3 inhibitors would reduce MGAT activity in HepG2 cells. Previous studies have demonstrated that both MGAT2 and MGAT3 isoforms are expressed in this cell line^23,40^. When crude mitochondrial membrane fractions isolated from HepG2 cells were treated with either the MGAT2 or MGAT3 inhibitors alone, there was only a ~ 24% and 35% decrease in MGAT activity, respectively (Fig. 1D). Treatment with both MGAT inhibitors together decreased MGAT activity by ~ 35% and did not have an additive effect. The modest reduction in MGAT activity in the presence of both MGAT2 and MGAT3 inhibitors suggested that the majority of in vitro MGAT activity must come from another enzyme. One possible candidate is DGAT1, which also has MGAT activity and can utilize 2-MG as a substrate to synthesize DG, at least in an in vitro assay^41^. We found that inhibition of DGAT1 with a small molecule inhibitor reduced the MGAT activity in HepG2 cells by ~ 90% (Fig. 1E). This finding provided additional evidence that DGAT1 accounted for most of the in vitro MGAT activity in HepG2 cells.

### Effect of MGAT inhibition on lipid droplet dynamics

**Figure 2:**
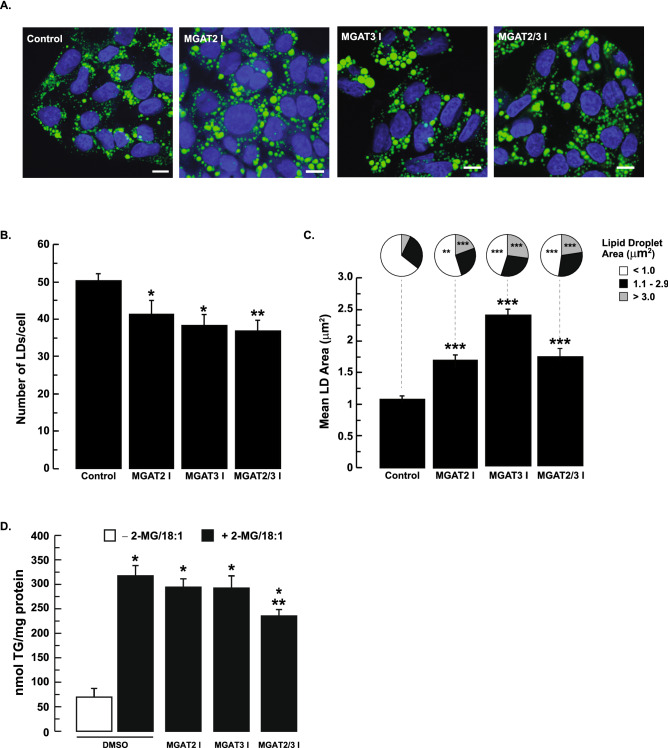
Inhibition of MGAT2 or MGAT3 alters lipid droplet morphology. (**A**) HepG2 cells were pre-incubated with 50 µM MGAT2 inhibitor or 100 µM MGAT3 inhibitor or both together for 2 h. Cells were then incubated with 0.5 mM oleic acid and 0.2 mM 2-MG for 12 h in the presence of the MGAT inhibitors. Cells were fixed and stained with Bodipy 493/503 and DAPI to visualize lipid droplets and nuclei, respectively. Scale bars, 10 µm. Lipid droplet number (**B**) and area (**C**) were quantified using ImageJ (National Institutes of Health, rsb.info.nih.gov/ij). Mean lipid droplet area per cell and lipid droplet number were calculated from 20 to 29 cells. Data are shown as means ± standard error. Size distribution of individual lipid droplets are shown as pie charts. Means were compared by ANOVA followed by Bonferroni Multiple Comparisons Test. *p < 0.05 versus DMSO-treated cells; **p < 0.01 versus DMSO-treated cells; ***p < 0.001 untreated versus DMSO-treated cells. (**D**) Intracellular TG levels in HepG2 cells are modestly reduced only when both MGAT2 and MGAT3 are inhibited. HepG2 cells were pre-incubated with the MGAT inhibitors or DMSO for 1 h. This was followed by incubating HepG2 cells with 0.2 mM 2-MG and 0.25 mM oleic acid (OA) in the presence of the MGAT inhibitors for 12 h. Cell extracts were prepared, and an aliquot was used for TG determination. *p < 0.001 *versus* DMSO-treated cells (without 2-MG/OA); **p < 0.001 versus DMSO-treated cells (with 2-MG/OA).

Inhibition of MGAT2 and MGAT3 only had a modest effect on in vitro MGAT activity. However, we explored the possibility that this might be sufficient to affect TG metabolism and alter lipid droplet morphology. To test this, HepG2 cells were pretreated with 50 µM MGAT2 inhibitor, 100 µM MGAT3 inhibitor or both inhibitors together. These concentrations of the inhibitors resulted in at least 80% inhibition of MGAT2 and MGAT3 (Fig. 1B). Lipid synthesis was then stimulated by incubating HepG2 cells with 0.5 mM oleic acid and 0.2 mM 2-MG in the presence of the MGAT inhibitors for 12 h. Analysis of lipid droplet morphology revealed that inhibition of MGAT2 or MGAT3, separately or together, decreased the number of lipid droplets per cell by 18%, 24% and 27%, respectively (Fig. 2A,B). Again, there was no significant additive effect when HepG2 cells were incubated with the inhibitors together. With the decrease in lipid droplet number, we observed an increase in lipid droplet size (Fig. 2A,C). The average area of lipid droplets in HepG2 cells treated with the MGAT2 inhibitor increased ~ 1.7-fold, while the average area of lipid droplets treated with the MGAT3 inhibitor increased 2.4-fold. For both MGAT2 and MGAT3 inhibition, we observed a decrease in the number of smaller lipid droplets (area < 1 µm^2^) which was accompanied by a corresponding increase in larger lipid droplets (area > 3 µm^2^) (Fig. 2A–C).

The changes in lipid droplet size and number suggested that intracellular TG levels might be altered. We hypothesized that if MGAT2 and/or MGAT3 contributed to TG synthesis in HepG2 cells, then inhibition of these enzymes should decrease intracellular TG levels. As expected, incubation of control HepG2 cells with 0.2 mM 2-MG and 0.25 mM oleic acid increased intracellular TG levels ~ fourfold compared to untreated cells (Fig. 2D). However, incubation of cells with either the MGAT2 or MGAT3 inhibitors had no effect on TG levels. In contrast, incubation of both MGAT2 and MGAT3 inhibitors together had a modest effect, reducing TG levels by ~ 26%.

### Effect of MGAT inhibition on TG synthesis and secretion from exogenously added 2-MG and oleic acid

To determine if MGAT2 and/or MGAT3 contributed to TG synthesis in HepG2 cells, radiolabeling experiments were performed in the presence and absence of the MGAT inhibitors.

**Figure 3:**
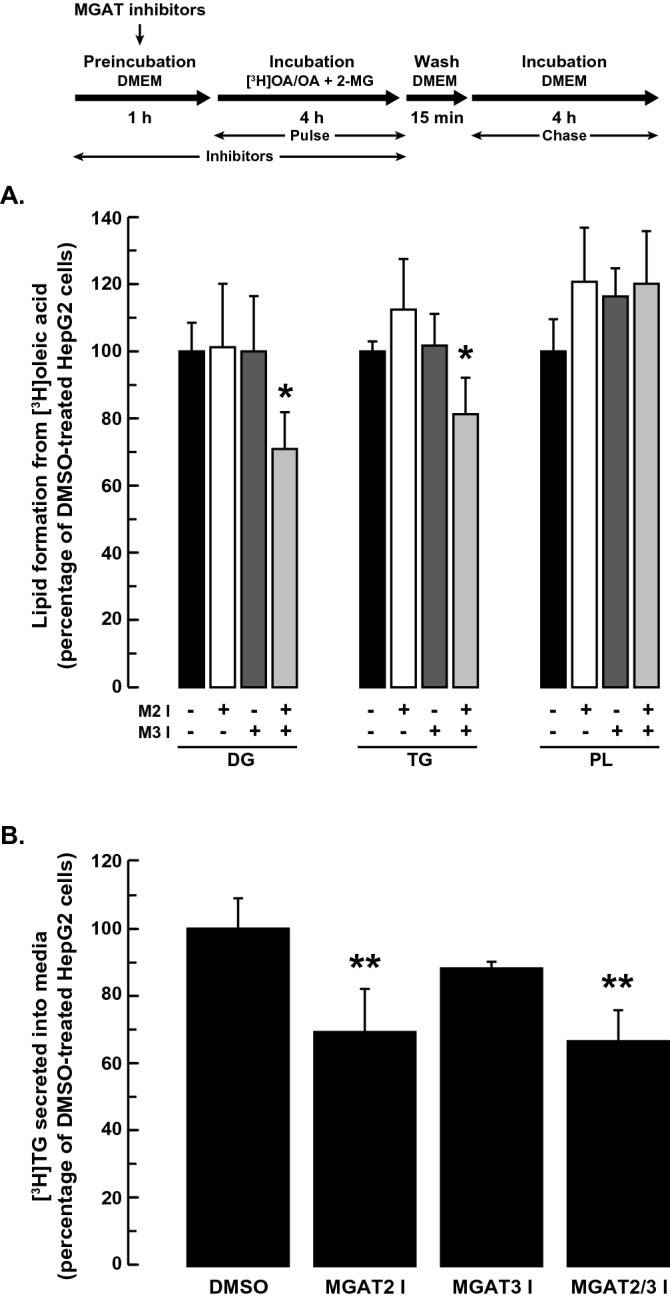
Effect of MGAT inhibition on lipid production from exogenous 2-MG and oleic acid. HepG2 cells were pre-incubated with or without the MGAT2 inhibitor (25 µM), MGAT3 inhibitor (50 µM) or both inhibitors together. After 1 h, 5 µCi [^3^H]OA, cold OA (0.25 mM final concentration) and 2-MG (0.2 mM final concentration) were added to the culture media and cells were incubated for a further 4 h. Cells were then washed and incubated an additional 4 h in DMEM. The incorporation of radioactivity into (**A**) TG, DG and PL in cells and (**B**) TG in media, was then determined. Values for DMSO-treated controls: DG (18.1 × 10^3^ dpm/mg cell protein), TG (332 × 10^3^ dpm/mg cell protein), PL (137 × 10^3^ dpm/mg cell protein), secreted TG (3.3 × 10^3^ dpm/mg cell protein). *p < 0.05 versus DMSO-treated cells; **p < 0.001 versus DMSO-treated cells.

We first examined if inhibiting MGAT2 and/or MGAT3 would alter TG synthesis from exogenously added 2-MG and oleic acid, both of which stimulate TG production^13,36^. When cells were incubated with exogenous oleic acid (containing [^3^H]oleic acid as a tracer) and 2-MG, inhibition of MGAT2 or MGAT3 had no apparent effect on the incorporation of radiolabeled oleic acid into DG or TG (Fig. 3A). When HepG2 cells were incubated with both inhibitors simultaneously, DG and TG production decreased by ~ 30% and 20%, respectively. The incorporation of [^3^H]oleic acid into phospholipids was not affected by inhibition of either MGAT, separately or together. Despite the lack of effect on intracellular DG and TG, MGAT2 inhibition did reduce TG secretion by ~ 30% (Fig. 3B). Inhibition of MGAT3 had no effect on TG secretion.

**Figure 4:**
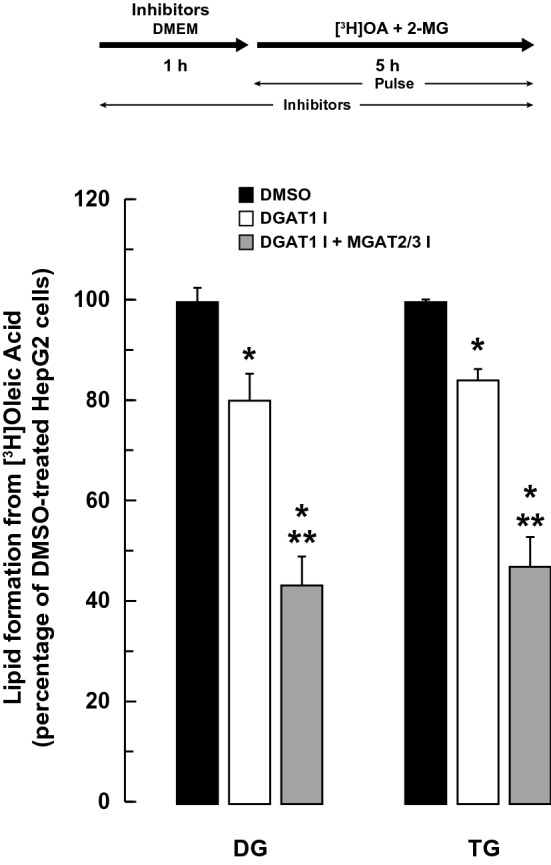
Effect of DGAT1 inhibition on lipid production from exogenous 2-MG and oleic acid. HepG2 cells were pre-incubated with or without the DGAT1 inhibitor (5 µM) or the DGAT1, MGAT2 (25 µM) and MGAT3 (50 µM) inhibitors together. After 1 h, 5 µCi [^3^H]OA, cold OA (0.25 mM final concentration) and 2-MG (0.2 mM final concentration) were added to the culture media and cells were incubated for a further 5 h. The incorporation of radioactivity into TG and DG in cells was then determined. Values for DMSO-treated controls: DG (22.3 × 10^3^ dpm/mg cell protein), TG (421 × 10^3^ dpm/mg cell protein). *p < 0.001 versus DMSO-treated cells; **p < 0.001 DGAT1 inhibitor versus DGAT1, MGAT2 and MGAT3 inhibitors together.

In an in vitro assay, DGAT1 accounted for the majority of the MGAT activity in HepG2 cells (~ 90%) (Fig. 1D,E). We next determined if the MGAT activity of DGAT1 contributed to TG production in intact HepG2 cells. When HepG2 cells were incubated with 2-MG and [^3^H]oleate in the presence of the DGAT1 inhibitor, DG and TG synthesis were only reduced by ~ 20% and ~ 16%, respectively (Fig. 4). The formation of radiolabeled DG and TG from 2-MG was reduced by ~ 40% when HepG2 cells were incubated with the DGAT1 inhibitor and both the MGAT2 and MGAT3 inhibitors together. These results suggest that, while DGAT1 accounts for most of the in vitro MGAT activity in HepG2 cells, the MGAT activity of DGAT1 only makes a small contribution to DG and TG synthesis when HepG2 cells are supplemented with 2-MG.

### Inhibition of MGAT2, but not MGAT3, reduced DG and TG production and TG secretion from exogenous oleic acid and glycerol

**Figure 5:**
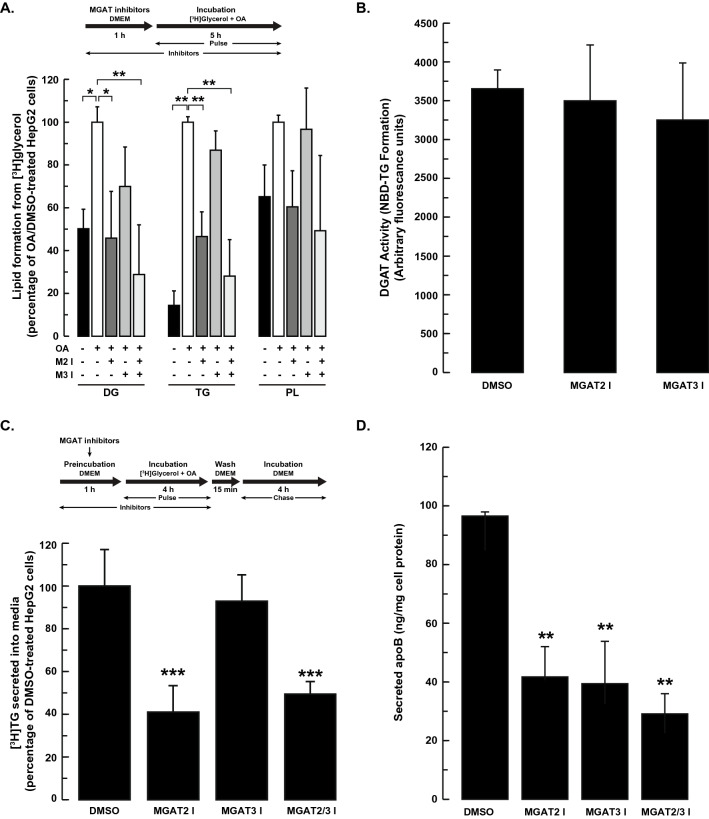
Inhibition of MGAT2, but not MGAT3, reduced DG and TG synthesis and TG secretion. (**A**) HepG2 cells were pre-incubated for 1 h with or without the MGAT2, MGAT3 or both inhibitors. Cells were then incubated with 5 µCi [^3^H]glycerol and 0.25 mM OA. After 5 h, cells were harvested and the incorporation of radioactivity into TG, DG and phospholipids was determined. (**B**) HepG2 cells were incubated with the MGAT2 (25 µM) or MGAT3 (50 µM) inhibitors for 5 h. Cells were harvested and DGAT activity in crude mitochondrial fractions was determined. Data are the mean ± standard deviation of two experiments performed in duplicate. (**C**) HepG2 cells were incubated with DMEM in the presence or absence of the MGAT2 or MGAT3 inhibitors (separately and together). After 1 h, cells were incubated with 5 µCi [^3^H]glycerol and 0.25 mM oleic acid for 4 h. Cells were then washed and incubated an additional 4 h in DMEM. The amount of [^3^H]TG secreted into the media was determined. Data are the mean ± standard deviation of two experiments performed in duplicate. Values for DMSO-treated controls: DG (1.9 × 10^3^ dpm/mg cell protein), TG (11.2 × 10^3^ dpm/mg cell protein), PL (30.2 × 10^3^ dpm/mg cell protein), secreted TG (0.89 × 10^3^ dpm/mg cell protein). (**D**) Effect of MGAT inhibition on apoB secretion. ApoB in the culture media was quantified by ELISA after incubation of HepG2 cells with 0.25 mM OA for 6 h in the presence of the MGAT inhibitors, as described in **A**. Data are the mean ± standard deviation of three independent experiments. *p < 0.01; **p < 0.001, ***p < 0.05.

We next wanted to determine if MGAT inhibition would influence TG metabolism in HepG2 cells supplemented with exogenously added [^3^H]glycerol and oleic acid. Incubation of HepG2 cells with exogenous 0.25 mM oleic acid increased the incorporation of [^3^H]glycerol into both DG (twofold) and TG (sevenfold) (Fig. 5A). Phospholipid synthesis trended higher but did not reach statistical significance. When HepG2 cells were incubated with the MGAT2 inhibitor, there was ~ 50% reduction in the incorporation of [^3^H]glycerol into both DG and TG. In contrast, the MGAT3 inhibitor had no apparent effect on DG or TG synthesis (Fig. 5A).

To rule out the possibility that the MGAT inhibitors were having a negative effect on cell health, cell viability was assessed by trypan blue exclusion. We found that neither the MGAT2 inhibitor nor the MGAT3 inhibitor had any detrimental effects on cell viability at the concentrations used in this study. Trypan blue staining demonstrated that cell viability was ~ 98% even after cells were incubated with either MGAT inhibitor (data not shown). The MGAT3 inhibitor has been shown to be well tolerated by HepG2 cells at concentrations higher than used in our experiments^35^. Less information is available regarding the MGAT2 inhibitor. A compound that is structurally related to the MGAT2 inhibitor that we used in our experiments has been used in a cell-based assay at similar concentrations without any indications of cell toxicity^42^. To determine if the decrease in DG and TG production was caused by non-specific inhibition of other acyltransferases, we measured DGAT activity after treatment of HepG2 cells with the MGAT inhibitors. Neither MGAT inhibitor had any affect on in vitro DGAT activity (Fig. 5B).

Because the MGAT2 inhibitor reduced TG synthesis in HepG2 cells, we tested the hypothesis that TG secretion would be reduced as well. HepG2 cells were pre-incubated with the MGAT2 inhibitor, MGAT3 inhibitor or both inhibitors together and then pulse labeled with [^3^H]glycerol and 0.25 mM oleic acid. We observed that TG secretion was decreased by ~ 60% in MGAT2 inhibitor treated cells (Fig. 5C). In contrast, the MGAT3 inhibitor had no effect.

We also analyzed the effect of MGAT inhibition on apolipoprotein B100 (apoB100) secretion. ApoB100 levels in the culture media were decreased by ~ 60% when either MGAT2 or MGAT3 were inhibited (Fig. 5D). However, simultaneous inhibition of both MGATs did not have a significant additive effect on apoB100 secretion. The decrease in both apoB100 and TG secretion suggested that MGAT2 inhibition led to a reduction in the number of VLDL particles secreted, but likely did not affect VLDL size or density. In contrast, inhibition of MGAT3 only decreased apoB100 secretion and had no apparent effect on TG secretion. This suggests that fewer VLDL particles were secreted but were likely larger with a reduced density.

### Inhibition of MGAT2 and MGAT3 reduced secretion of stored TG

**Figure 6:**
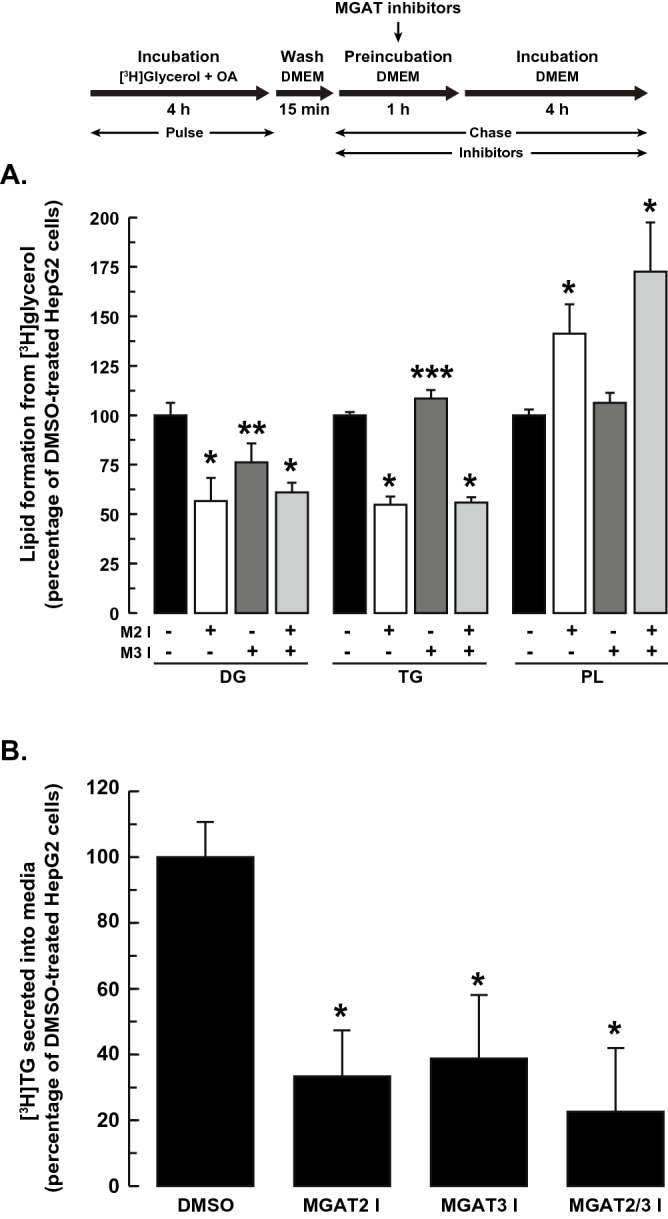
MGAT2 contributes to TG re-esterification. HepG2 cells were incubated with DMEM containing 5 µCi [^3^H]glycerol and 0.25 mM oleic acid for 4 h. The radioactivity was washed away, and cells were then incubated with the MGAT inhibitors, separately or together. After 1 h, the media was replaced with fresh DMEM (containing MGAT inhibitors) and incubated for another 4 h. The incorporation of radioactivity into (**A**) intracellular lipids (TG, DG and PL) and (**B**) secreted TG was then determined. Values for DMSO-treated controls: DG (3.6 × 10^3^ dpm/mg cell protein), TG (82.0 × 10^3^ dpm/mg cell protein), PL (57.2 × 10^3^ dpm/mg cell protein), secreted TG (0.90 × 10^3^ dpm/mg cell protein). *p < 0.001 versus DMSO-treated cells; **p < 0.01 versus DMSO-treated cells; ***p < 0.05 versus DMSO-treated cells.

We next sought to determine if MGAT2 and/or MGAT3 participated in the lipolysis/re-esterification cycle of stored TG and its secretion. We performed pulse-chase radiolabeling experiments in which the TG pool in HepG2 cells was first radiolabeled with [^3^H]glycerol in the presence of 0.25 mM oleic acid. Cells were then incubated with the MGAT inhibitors for 5 h, after which the amount of radioactivity in DG, TG and phospholipids was determined. Inhibition of both MGAT2 and MGAT3 reduced the amount of [^3^H]glycerol in DG by ~ 43% and 24%, respectively, and suggested that both enzymes could participate in the re-esterification of 2-MG in hepatocytes (Fig. 6A). In contrast, the resynthesis of TG was only reduced in MGAT2-inhibitor treated cells, which suggested that the G3P pathway might compensate when MGAT3, but not MGAT2, is inhibited. Inhibition of MGAT2 also resulted in increased incorporation of [^3^H]glycerol into phospholipids (Fig. 6A). The likely reason for this is that fatty acids that could not be used for TG production were instead redirected to phospholipids.

The decrease in TG re-esterification when MGAT2 was inhibited suggested that TG secretion would be affected as well. Indeed, inhibition of MGAT2 markedly reduced TG secretion by ~ 67% (Fig. 6B). This result suggested that MGAT2 could have a prominent role in the re-esterification of 2-MG to produce a pool of TG that can be utilized for VLDL assembly and secretion. Unexpectedly, TG secretion was also reduced by ~ 60% when MGAT3 was inhibited (Fig. 6B). This was despite the observation that inhibition of MGAT3 only affected re-esterification of 2-MG, but not DG (Fig. 6A). Incubation of HepG2 cells with both inhibitors together did not have an additive effect on TG secretion.

## Discussion

TG synthesis in the liver has primarily been attributed to the glycerol 3-phosphate pathway. However, the presence of MGAT enzymes in the liver suggested that the MGAT pathway might also contribute to some TG synthesis in this tissue. In this study, we utilized pharmacological inhibitors of MGAT2 and MGAT3 to explore their relative contributions to TG synthesis and secretion in HepG2 cells. We found that inhibition of either MGAT2 or MGAT3 in HepG2 cells modestly reduced in vitro MGAT activity and had little effect on intracellular TG levels. However, we did observe changes in lipid droplet dynamics. Inhibition of either MGAT2 or MGAT3 resulted in a decrease in intracellular lipid droplet number that was accompanied by a corresponding increase in lipid droplet size. The effect was somewhat more pronounced under conditions of MGAT3 inhibition compared to that of MGAT2. Only when both enzymes were inhibited together was there a small decrease in intracellular TG. This is different from mice that were chronically treated with an MGAT2 inhibitor that had reduced liver MGAT activity and TG content^33,43^. The lack of effect on TG levels in MGAT2 inhibitor-treated HepG2 cells may be due to compensation by MGAT3, which is not present in mouse liver^24^.

That intracellular TG levels did not decrease under conditions of MGAT2 or MGAT3 inhibition suggested that neither of these enzymes had an appreciable role in TG metabolism in the liver. Furthermore, inhibition of MGAT2 or MGAT3 individually had no effect on DG/TG synthesis when HepG2 cells were provided with exogenous 2-MG and fatty acids, substrates that MGAT enzymes would typically utilize in the small intestine. Only when both enzymes were inhibited together was there a modest effect on DG and TG production from 2-MG. Experiments that we performed with a DGAT1 inhibitor showed that DGAT1 accounted for ~ 80–90% of the MGAT activity in vitro in HepG2 cells which was consistent with previous findings^23^. However, inhibition of DGAT1 only modestly decreased both DG and TG production in HepG2 cells by ~ 20% when they were supplemented with 2-MG. These observations suggested that the MGAT activity of DGAT1 only makes a minor contribution to DG and TG synthesis. In the small intestine, which also has an active MGAT pathway, there was no apparent decrease in DG or TG in the small intestine of *Dgat1*-deficient mice fed a high-fat diet^44^.

Inhibition of MGAT2 did result in a marked decrease in DG and TG production and TG secretion when HepG2 cells were incubated with [^3^H]glycerol and fatty acids. This finding suggested that MGAT2 in HepG2 cells could utilize 2-MG derived from TG that was initially produced from the glycerol 3-phosphate pathway. Alternatively, there is some evidence suggesting that glycerol can be directly acylated to produce monoacylglycerol^45^. Whether or not this is 2-MG or 1(3)-MG is not known.

HepG2 cells have been widely used as a model of human hepatocytes to study lipoprotein metabolism. However, although they have the capacity for TG synthesis, the ApoB100 particles that they secrete are not well lipidated compared to those produced by the liver^46,47^. This appears to be caused by hyperactive MEK-ERK signalling which impairs the transfer of TG from cytosolic stores to the microsomal pool that is utilized for the lipidation of apoB100^47–49^. Despite this defect and impaired VLDL secretion, our pulse/chase experiments suggested that, in HepG2 cells, MGAT2 can utilize 2-MG derived from the turnover of intracellular TG that is part of the lipolysis/re-esterification cycle of hepatic TG. The synthesis of DG from 2-MG may have an energy conserving function as glycerol kinase, which consumes ATP, would be bypassed.

**Figure 7:**
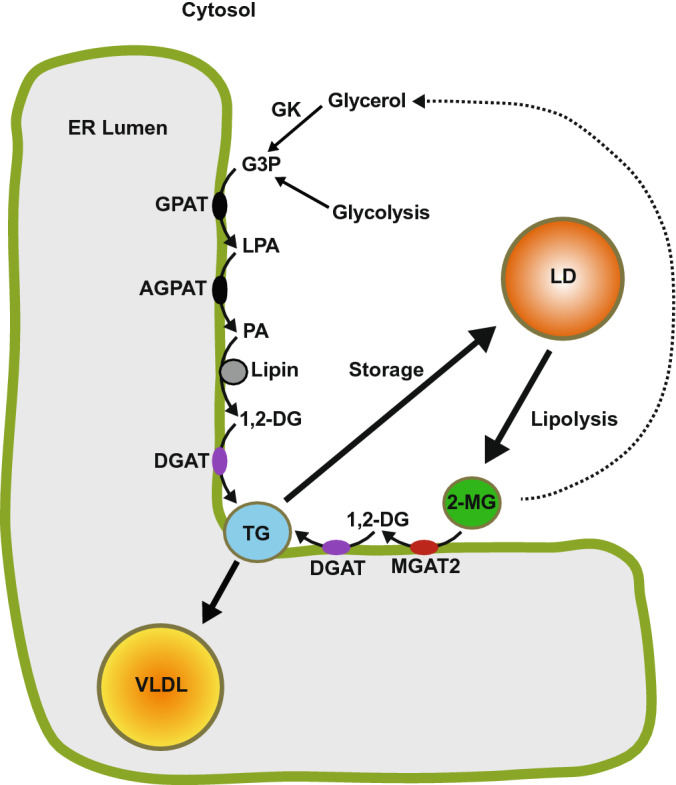
MGAT2 has a role in the lipolysis/re-esterification of stored TG in hepatocytes. TG synthesis at the endoplasmic reticulum begins with glycerol 3-phosphate (G3P) from glycolysis or by the phosphorylation of glycerol by glycerol kinase (GK). G3P undergoes two successive acylation reactions first producing 1-acylglycerol 3-phosphate (LPA) and then phosphatidate (PA) which is dephosphorylated to 1,2-diacylglycerol (1,2-DG). DGAT catalyzes a third esterification reaction producing TG that is stored in cytosolic lipid droplets (LDs) and can subsequently be used for VLDL assembly. VLDL acquire their TG via the lipolysis/reesterification of preformed TG stored in LDs. Lipases hydrolyze LD TG generating 2-monoacylglycerol (2-MG). Monoacylglycerol acyltransferase-2 (MGAT2) uses this 2-MG as a substrate producing 1,2-DG and subsequently TG, which is utilized for VLDL assembly at the ER. Some glycerol is also likely generated from the complete hydrolysis of TG, which can re-enter the glycerol 3-phosphate pathway. GPAT, glycerol 3-phosphate acyltransferase; AGPAT, 1-acylglycerophosphate acyltransferase.

Although the liver TG pool rapidly turns over, most of the released fatty acids are re-esterified to TG with only a small fraction of them incorporated into VLDL-TG^50^. Approximately 70% of TG secreted from hepatocytes comes from cytosolic TG stores that undergoes lipolysis/re-esterification^51,52^. It has been suggested that stored TG is only partially hydrolyzed to 2-MG, which theoretically could then be used as a substrate by MGAT2 to resynthesize DG and then TG^52^. An analogous system exists in the small intestine, where ~ 50% of stored TG is hydrolyzed to 2-MG which is then re-esterified to TG and incorporated into chylomicrons^53^. Our own experiments, using [^3^H]glycerol to radiolabel lipids, demonstrated that MGAT2 utilized 2-MG derived from the glycerol 3-phosphate pathway, presumably generated from the breakdown of stored TG (Fig. 7).

Our experiments demonstrated that in HepG2 cells, MGAT2 had a more prominent role in both TG synthesis and secretion than MGAT3. This was somewhat unexpected since MGAT2 protein could not be detected in human liver despite its high level of expression^23^. In the same study, a full-length *MOGAT1* transcript could not be amplified from several tissues, including liver, suggesting that MGAT3 was the prominent MGAT enzyme in this tissue. However, knockdown of *MOGAT3* in HepG2 cells only reduced MGAT activity by ~ 20%^23^. These findings imply that MGAT2 may have a more pronounced role in hepatic TG metabolism, despite its low abundance.

The general lack of effect on TG metabolism in HepG2 cells treated with the MGAT3 inhibitor was unexpected. MGAT3 does appear to be capable of promoting TG synthesis in intact cells as a previous study demonstrated that overexpression of MGAT3 in HepG2 cells increased DG and TG labeling from [^3^H]glycerol^23^. In the same study, knock down of MGAT3 expression in HepG2 cells using RNAi interference reduced MGAT activity by ~ 30%, which agreed with our findings. However, no data from experiments performed in intact HepG2 cells were reported. In conclusion, the contribution of MGAT3 to TG metabolism remains largely unknown.

A limitation of this study is that HepG2 cells are not a surrogate for human hepatocytes. Indeed, in addition to the alterations in lipid metabolism already described, there are extensive differences between the global transcriptomes and proteomes of HepG2 cells and primary human hepatocytes^54,55^. Future studies of MGAT2 and MGAT3 in primary human hepatocytes will shed more light on the roles that these enzymes have in hepatic lipid metabolism.

## Materials and methods

### Cell culture and transfection

HepG2 cells (American Type Tissue Culture Collection) were cultured in Dulbecco's Modified Eagle Medium (DMEM): Nutrient Mixture F-12 with 10% fetal bovine serum in a 37 ^◦^C incubator with 5% CO_2_. To stimulate lipid synthesis, cells were incubated with 0.25 or 0.5 mM oleic acid complexed to 0.67% fatty acid–free bovine serum albumin. Both of these concentrations of oleic acid increase intracellular TG levels in HepG2 cells several-fold^56^. Maximal stimulation of apolipoprotein B secretion in HepG2 cells has been shown to occur at 0.1 mM and stays relatively constant up to 0.8 mM^57^. Alternatively, HepG2 cells were incubated with 0.2 mM 2-oleoylglycerol (2-MG). 2-MG (Santa Cruz Biotechnology: sc-213888) was dissolved in 0.5% ethanol (v/final volume) and then dispersed in DMEM containing 100 μM fatty acid–free bovine serum albumin^58^. The mixture was incubated at 37 °C for 1 h with shaking and then incubated with cells. HepG2 cells were incubated with the MGAT2 inhibitor (Cpd24d) (Sigma: 538437) and/or the MGAT3 inhibitor (PF-06471553, Pfizer Global Medical Grant Program) and/or the DGAT1 inhibitor (PF-04620110) (Sigma: PZ0207), as indicated^34,35,39^. Inhibitors were dissolved in DMSO.

HEK-293T (American Type Tissue Culture Collection) were cultured in DMEM and 10% fetal bovine serum in a 37 °C incubator with 5% CO_2_. For cell transfections, 20 µg of plasmid DNA (FL-MGAT2, FL-MGAT3 or FL-DGAT1) was incubated with 430 µL of 0.15 M NaCl and 120 µL of 0.1% polyethylenimine (pH 7.0) for 10 min at room temperature. The transfection mixture was then added dropwise to a 100-mm culture dish containing 10 mL of DMEM with 10% fetal bovine serum and cells at ~ 50% confluence. After 4 h, the medium was removed, and cells were washed and re-fed with fresh medium. Cells were harvested and used for experiments 48 h after transfection.

### Cell viability

After MGAT inhibitor treatments, HepG2 cells were washed with PBS, released from culture dishes with 0.25% trypsin and harvested in DMEM. Cell were diluted 1:1 (v/v) with 0.4% trypan blue. An aliquot was placed on a hemocytometer and examined under a light microscope. Viable cells were counted by their ability to exclude trypan blue.

### Isolation of crude mitochondrial membrane fractions

Washed cell pellets were resuspended in 50 mM Tris–Cl (pH 7.6)/250 mM sucrose. Cells were disrupted by 25 needle passages through a 27-gauge needle. Cell debris and nuclei were pelleted by centrifugation at 1000 × *g* for 2 min. The supernatant was centrifuged at 10,000 × *g* for 10 min at 4 °C to pellet crude mitochondria (mitochondria and MAM) which were resuspended in 50 mM Tris–Cl (pH 7.6)/250 mM sucrose. Crude mitochondrial membranes (20–50 µg of protein) were used to measure MGAT/DGAT activities after MGAT inhibitor treatments.

### MGAT and DGAT activity assays

MGAT activity was determined by measuring the formation of N-[(7-nitro-2–1,3-benzoxadiazol-4-yl)-methyl] amino (NBD)-DG from NBD-palmitoyl-CoA^36^. The reaction mixture contained 100 mM Tris–Cl (pH 7.4), 20 mM MgCl_2_, 0.625 mg/mL BSA, 200 µM 2-monooleoylglycerol, 25 µM NBD-palmitoyl-CoA (Avanti Polar Lipids: 810705P) and 25–50 µg of protein sample, in a final volume of 200 µL. Samples were incubated at 37 °C for 10 min and reactions were terminated by the addition of chloroform:methanol (2:1). Lipids were extracted and separated by thin layer chromatography in diethyl ether/hexane/methanol/acetic acid (55:45:5:1, v/v/v/v). NBD-DG was detected with a VersaDoc 4000 molecular imaging system (Bio-Rad Laboratories, Inc.) and fluorescence was quantified with Quantity One software (Bio-Rad Laboratories, Inc.). For some experiments, protein samples were incubated with a DGAT1 inhibitor prior to performing MGAT assays.

DGAT assays were performed as described for the MGAT assays, except that 200 µM 1,2-dioleoylglycerol in acetone was used as the acyl acceptor. The product of the reaction, NBD-TG, was analyzed by thin layer chromatography as described above.

### Radiolabelling

[2-^3^H]glycerol (25 Ci/mmol) (ART-0188B) and [9,10-^3^H(N)] oleic acid (60 Ci/mmol) (ART-0198) were from American Radiolabeled Chemicals. Cellular lipids were radiolabeled by incubating cells with either [^3^H]oleic acid (5 µCi) and 200 µM 2-MG or [^3^H]glycerol (5 µCi) and 0.25 mM oleic acid in serum-free DMEM. After incubation for the indicated times, lipids were extracted from cell lysates or culture media by the method of Bligh and Dyer^59^ with ClCH_3_:MeOH (2:1, v/v), separated by thin layer chromatography on silica gel G-60 thin layer chromatography plates in the solvent system hexane:diethyl ether:acetic acid (80:20:1, v/v/v). Lipids were visualized by staining with iodine vapors and the radioactivity in TG, DG and PL was determined by liquid scintillation counting. Results were normalized to intracellular protein content.

### Intracellular TG determination

Intracellular TG levels were determined using the Triglyceride Assay Kit (Abcam: Ab65336) following the manufacturers directions. Measurements were made within the linear range of the assay. Results were normalized to intracellular protein.

### Determination of secreted apolipoprotein B100 (ApoB)

HepG2 cells were seeded into 12-well-plates and grown to 70% confluence. Cells were incubated for 1 h with the MGAT inhibitors in 1 mL DMEM (25 µM MGAT2, 50 µM MGAT3, or both inhibitors together). Cells were then incubated in 1 mL DMEM containing the MGAT inhibitors and 0.25 mM oleic acid for 6 h. ApoB levels in the media were quantified using a Human ApoB ELISA kit (Invitrogen: EH34RB) following the manufacturers directions. Results were normalized to cell protein.

### Fluorescence microscopy

Cells were grown in 6-well dishes containing glass coverslips. Cells were washed with PBS, fixed with 4% paraformaldehyde in PBS for 10 min and permeabilized with 0.2% Triton X-100 for 2 min. Lipid droplets were visualized by staining fixed cells with BODIPY 493/503 (Invitrogen: D3922). Coverslips were mounted on glass slides with a drop of mounting medium containing DAPI (ThermoFisher: P36966) to detect nuclei. All manipulations were performed at room temperature. Confocal images were acquired using a Zeiss LSM700 laser-scanning confocal microscope. Images were processed using Fiji^60^.

### Statistical analyses

Unless otherwise indicated, values presented are means ± standard deviations. Means were compared by analysis of variance (ANOVA) followed by the Tukey test. All experiments were performed at least three times unless otherwise indicated.

## Supplementary Information

Supplementary Information.

Supplementary Figure 1.
