# Supplementary material for: The monoacylglycerol acyltransferase pathway contributes to triacylglycerol synthesis in HepG2 cells
Source: Sci Rep. 2022 Mar 23;12:4943. doi: 10.1038/s41598-022-08946-y (PMC8943211; doi:10.1038/s41598-022-08946-y)
Supplement: Supplementary file 1 — Supplementary Information. [file 41598_2022_8946_MOESM1_ESM.docx]

Supplementary Figure 1. Overexpression of FL-MGAT2, FL-MGAT3 and FL-DGAT1 in HEK-293T cells. Crude mitochondrial membrane fractions isolated from HEK-293T cells expressing FL-MGAT2, FL-MGAT3 or FL-DGAT1 were separated by SDS-PAGE and immunoblotted with anti-FLAG (α-FLAG) and anti-mitochondrial HSP70 (α-HSP70) antibodies. The red boxes indicate the areas used in Figure 1A.
